# Cost Analysis of Endoscopic Submucosal Dissection for the Treatment of Colorectal Lesions in China

**DOI:** 10.1155/2019/6983896

**Published:** 2019-03-26

**Authors:** Ning Cui, Yu Zhao, Honggang Yu

**Affiliations:** Department of Gastroenterology, Renmin Hospital of Wuhan University, Wuhan, China

## Abstract

**Aim:**

The aim of the study was to evaluate costs associated with colonic endoscopic submucosal dissection (ESD) for treatment of colorectal cancer.

**Methods:**

The study is a retrospective analysis of data on 395 patients treated by colonic ESD.

**Results:**

The operation, consumable items, and medication accounted for 71% of the total costs for colonic ESD treatment. Medication and consumable items' costs were higher if lesions occurred in the transverse colon and right hemicolon compared to the left hemicolon. Medication, consumable items, and total costs were higher for larger lesions. Lesion numbers and carcinoma were associated with higher medication, consumable items, operation, and total costs. Positive surgical margins and complications of hemorrhage or perforation were positively correlated with higher costs for medication, consumable items, and total costs.

**Conclusion:**

Labor costs for doctors and nurses remain low in China. Costs for medication and consumable items were higher for treatment involving the transverse colon or right hemicolon (vs. the left hemicolon), larger lesions, carcinoma, and a positive surgical margin. A benchmark cost estimate for ESD treatment including 4 days of postoperative hospitalization was determined to be approximately 5400 USD.

## 1. Introduction

Colorectal cancer is among the top three causes of cancer mortality and is commonly diagnosed in both Western and Eastern nations, including China [1]. Based upon data collected in the USA and Japan, current levels of colorectal cancer reflect a sharp increase in incidence. High costs of colorectal cancer treatment have, accordingly, dramatically increased the global healthcare economic burden [2]. Early stage colorectal cancer can be effectively treated using advanced endoscopic procedures, including endoscopic submucosal dissection (ESD) [3]. ESD is a very promising approach that delivers favorable short-term outcomes. Multiple procedures can be performed simultaneously. ESD is minimally invasive and results in high radical cure rates, removal of pathological tissues, and rapid recovery [4]. Compared to conventional endoscopic procedures such as endoscopic mucosal resection, ESD exhibits higher* en bloc* resection rates and lower recurrence rates for early stage gastric and colorectal cancer [5, 6]. At the present time, optimized cost-benefit ratios for hospitalization time with respect to pre-ESD preparation and post-ESD complications remain undefined. To fill this knowledge gap, we performed a retrospective analysis of data collected on nearly 400 patients in order to examine the cost-effectiveness of colonic ESD.

## 2. Materials and Methods

### 2.1. Patient Cohort

The study is based on retrospective analysis of data from 395 patients undergoing colonic ESD between January 2015 and December 2017 at the Department of Gastroenterology, Renmin Hospital of Wuhan University, China.

### 2.2. Lesion and Operation Characteristics

Tumor size (13.9 ± 2.7 mm, median 24 mm, and range from 6 to 105 mm) was included. Pathologies of the neoplasm were summarized as polyp (hyperplastic, 29, 7.3%; inflammatory, 52, 13.2%), adenoma, carcinoid, and carcinoma. Moreover, macroscopic morphology of the lesions (LST types: LST-G, 27, 6.8%; LST-NG, 37, 9.4%) was presented as well (type 0-IIc 31, 7.8%; 0-IIa+IIc, 26, 6.6%; type 0-I, 7, 1.8%). The operators consisted of junior operators and senior professors. On one hand, there were three well-trained junior doctors (trained for more than three years and operated upon more than one hundred pig stomachs and colons) who participated in the beginning steps for 72 cases that were supervised by senior professors. On the other hand, four senior professors who practiced colonic ESD for more than 500 cases and more than ten years were enrolled. The indications strictly followed the colonic ESD guidelines including polys/LST bigger than 2 cm, a submucosal tumor, carcinoid or rectal carcinoid smaller than 2 cm, and early carcinoma. The operations (techniques and procedures) were conducted according to colonic ESD guidelines, such as lesion observation, marking, submucosal injection, incision, dissection, and wound management. Complications (intraprocedural and postoperative) were involved as well.

### 2.3. Cost Analysis

The analyzed colonic ESD patient medical costs spanned hospital admission to discharge. Medical costs were categorized as direct, indirect, and intangible. Direct costs were related to treatment and patient care including admission, medical procedures, consumable items, medications, and nursing. Indirect costs were those ascribed to disease-related morbidity and mortality. Intangible costs encompassed nonfinancial outcomes such as pain and mental suffering. Direct medical charges were obtained from hospitalized patient charge lists (CHIS 7.0, Founder International Co., Ltd., China).

### 2.4. Statistical Analysis

Statistical analysis was performed using SPSS version 17.0 (SPSS, Chicago, IL, USA). Numerical data are presented as mean ± SD. Student's* t*-test was used for comparison between groups.* P*<0.05 was considered statistically significant.

## 3. Results

Table 1 presents clinical features and proximal outcomes for 395 patients (214 male, 185 female) between the ages of 19 and 83 years undergoing colonic ESD. The procedure duration ranged from 11 to 172 min. Tumors varied in diameter from 6 to 105 mm and were variably located from the rectum to the ascending colon. Tumors comprised polyps (20.5%), adenomas (45.3%), carcinoids (22.0%), and carcinomas (12.2%). Nine complications arose during ESD and 56 were recorded after the procedure. Postprocedure and total duration of hospital stay were also recorded.

**Figure 1:**
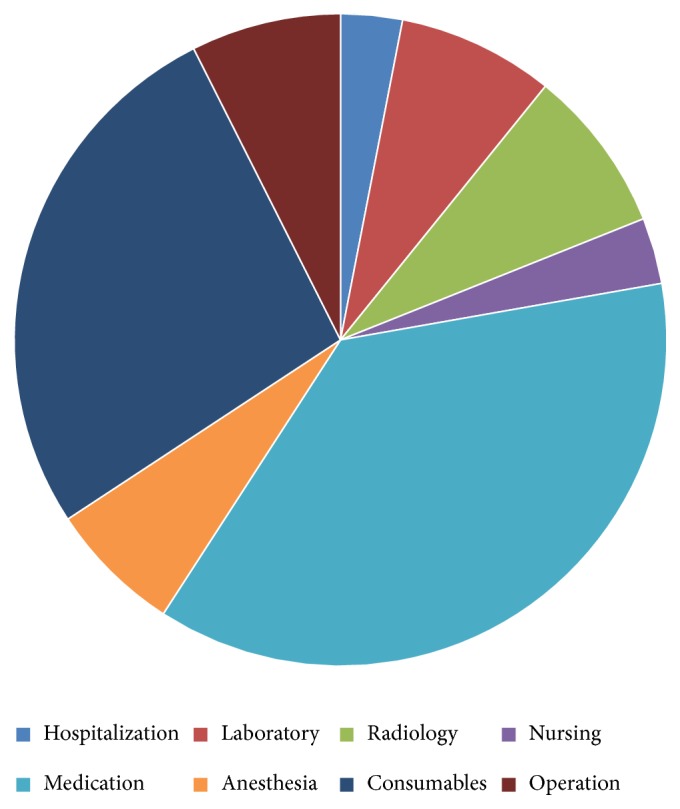
Distribution of medical costs during hospitalization.

We divided costs into several categories: hospitalization, laboratory, imaging, nursing, medication, anesthesia, consumable items, and operation. The hospital financial system precluded more specific cost stratification in this study. Total costs were divided into those covered by National Health Insurance (NHI) and those paid by the patients themselves. Operation, medication, and consumable items' costs accounted for 71% of the total costs for colonic ESD (Table 2 and Figure 1).

**Figure 2:**
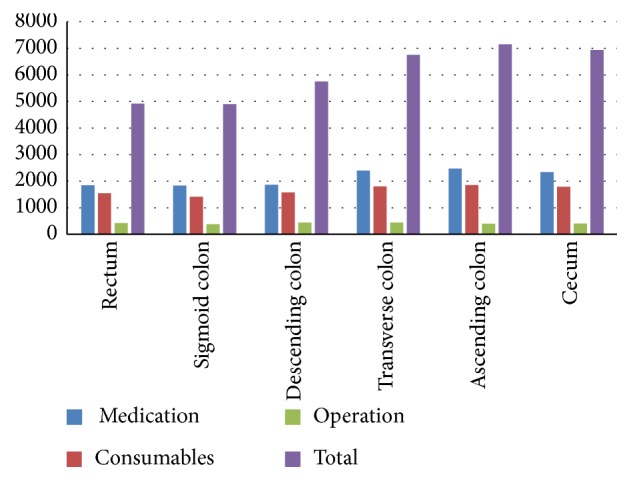
Distribution of major costs based on lesion location.

We examined relationships between cost and lesion location. Medication and consumable items' costs were higher for lesions in the transverse colon and right hemicolon (>2300 USD) than for lesions in the left hemicolon (<1900 USD). Total costs showed the same trend, with costs exceeding 6700 USD for lesions in the transverse colon and right hemicolon but less than 5800 USD for lesions in the left hemicolon. No major differences in operation costs were observed for lesions in different locations (Table 3 and Figure 2).

**Figure 3:**
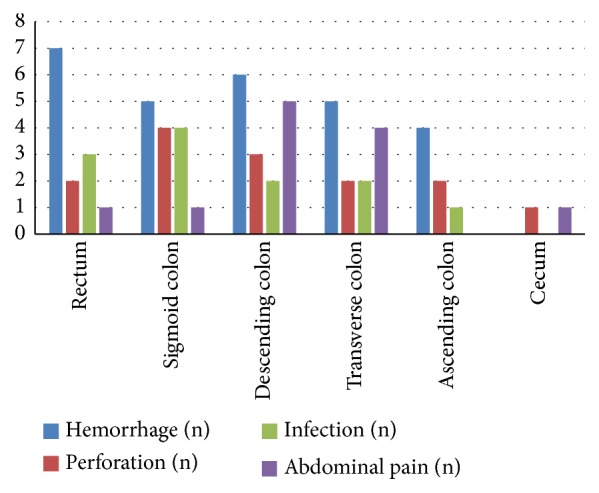
Complications based on lesion location.

To determine the reasons for differential costs associated with different lesion locations, we examined the relationship between location and postprocedure complications. No significant differences were found for hemorrhage, perforation, or infection between different locations (*P*>0.05). However, abdominal pain occurred significantly more frequently following procedures for lesions of the ascending and transverse colon than for other locations (*P*<0.05) (Table 4 and Figure 3).

**Figure 4:**
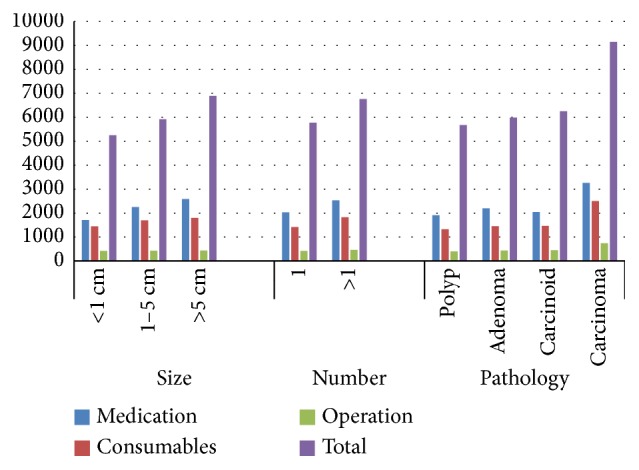
Distribution of main costs based on lesion size, number, and pathology.

We observed that costs for medication and consumable items and total costs were significantly higher for lesions in the size ranges of 1–5 cm and >5 cm than for lesions <1 cm (*P*<0.05). Operation costs, however, did not significantly differ according to lesion size (*P*> 0.05). The presence of multiple lesions (>1) was significantly correlated with higher costs for medication, consumable items, and operation and total costs compared to patients with a single lesion (*P*< 0.05). Carcinoma was associated with significantly (*P*< 0.05) higher costs for medication, consumable items, and operation and total costs compared to other pathologies (Table 5 and Figure 4).

**Figure 5:**
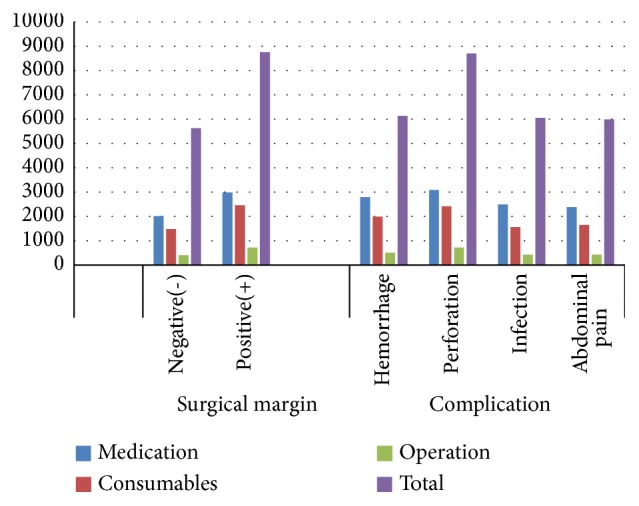
Distribution of main costs based on surgical margins and complications.

Stratifying patients based on operation characteristics, we observed that positive surgical margins were associated with significantly higher medication, consumable items, and total costs compared to negative surgical margins (*P*< 0.05). With respect to complications, hemorrhage and perforation were associated with significantly higher medication, consumable items, and total costs compared to complications of infection and abdominal pain (Table 6 and Figure 5).

**Figure 6:**
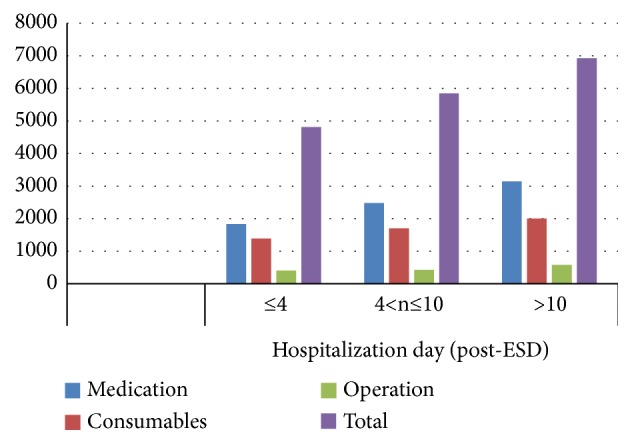
Distribution of costs based on hospitalization days after the procedure.

To better understand differential costs associated with duration of hospitalization, we analyzed the occurrence of post-ESD complications. Total post-ESD complications comprised hemorrhage (6.84%, 27/395), perforation (3.54%, 14/395), infection (3.04%, 12/395), and abdominal pain (3.04%, 12/395). The incidence of complications was significantly higher within 4 d after the procedure (87.7%) than at 4 d (12.3%) (Table 7). We examined costs relative to length of hospitalization (≤4 d, 4–10 d, and >10 d). Hospitalization of ≤4 d and >10 d was associated with the lowest and highest, respectively, medication, consumable items, and total costs (*P*< 0.05). Hospitalization of >10 d was correlated with significantly higher operation costs (Table 8 and Figure 6).

Lastly, in order to clarify the relationship between costs and outcomes, the costs have been analyzed for defined outcomes of colorectal ESD. More detailedly, the costs of uncomplicated curative ESD of cancer (and HGIN), uncomplicated noncurative ESD of cancer, and complicated ESD of cancer (and of other benign lesions) were analyzed, respectively (Table 9).

## 4. Discussion

Minimally invasive ESD is a promising technique for treatment of digestive tract diseases, especially polyps, adenoma, and early stage carcinoma [7]. Most well-trained gastrointestinal endoscopic physicians can correctly perform ESD operations [8]. Colonic ESD will certainly become more prevalent due to higher patient satisfaction, more comprehensive indications, shorter hospitalization times, and lower costs compared to traditional laparotomy surgery [9]. In China, NHI covers some costs associated with colonic ESD, except for some consumable items and medication [10]. The present study helps to clarify some cost issues related to colonic ESD treatment in China. More specifically, we examined costs of clinical characteristics including lesion location, size, number, and pathology; surgical margin and complications; and hospital stay after the procedure. Costs for medication and consumable items together accounted for >60% of total costs. Operation and nursing costs combined comprised only 10% of total costs, indicating that physician and nurse labor costs in China remain low.

Nearly 90% of complications occurred within 4 d after the procedure. With respect to lesion location, abdominal pain occurred more often when lesions were located in the ascending and transverse colon. This might be due to poor gas release from high positions of the colon, with the pneumogastric nerve only exiting above the splenic flexure [11, 12]. When operating on high colonic positions surgeons generally take more time and use more consumable items (such as metal clips) to avoid complications such as hemorrhage or perforation [13]. In contrast, studies showed that, even in the right colon, the complication rate should not be increased for ESD on the professional level [14, 15]. This in turn requires patients to stay longer in hospital for postoperative observation [16]. This could contribute to higher costs for medication and consumable items and total costs for high-position colon ESD. ESD to treat larger lesions also requires more consumable items, as well as more medication to promote wound healing and patient recovery [17, 18]. We made similar observations with respect to lesion number. Some carcinoma patients received surgical and additional oncology treatment, and thus costs for these patients were relatively high [19]. Positive surgical margins were associated with higher medical, consumable items, operation, and total costs [20, 21]. Patients with post-ESD complications of hemorrhage and perforation had higher costs associated with repeat endoscopy and/or surgical treatment [22, 23]. Based on defined outcomes, complicated colonic ESD of cancer occupied the highest position for costs of all aspects on account of more usage of medication and consumables, more complex operations and multiple therapies intervention, and so on [24]. However, the uncomplicated operations had lower medication, consumable items, operation, and total costs comparing to the complicated ones. The reason might be effective coaching and cost controlling in the process. Even when well-trained junior physicians participated in some parts of an ESD operation, senior professors supervised and step-by-step guided the whole procedure [14, 25]. Further efforts (randomized control trial) could be focused on costs saving of different treatment choices, such as cost savings of professional colorectal ESD as compared to elective laparoscopic operation or surgery for a specific indication [26]. Our study indicated only 10.6% malignant lesions were resected by colorectal ESD. Therefore, more profound thinking should be concentrated on the endoscopic diagnosis accuracy and indication criteria selection. Cheaper techniques (e.g., EMR) should be performed for benign neoplasia [27]. For instance, small and rectal carcinoid could be easily resected with much cheaper procedures, such as rubber band EMR or UEMR [28]. Our analysis suggests benchmark costs for colonic ESD of approximately 400 USD for the procedure itself and a total treatment cost of 5000 USD. The current study retrospectively examined costs for 395 patients treated at a single center. Additional prospective multicenter studies will be needed to fully evaluate issues of cost-effectiveness.

## Figures and Tables

**Table 1:** Clinical features and short-term outcomes for 395 patients treated with colonic ESD.

Age (yr)	51.9 ± 6.7 (19–83)
Gender	
Male (214)	
Female (181)	
Procedure time (min)	47.7 ± 5.0 (11–172)
Tumor size (mm)	13.9 ± 2.7 (6–105)
Tumor location	
Distance from anus (cm)	36.2 ± 2.9 (5–72)
Pathology	
Polyp	81 (20.5%)
Hyperplastic	29 (7.3%)
Inflammatory	52 (13.2%)
Adenoma	185 (46.9%)
Carcinoid	87 (22.0%)
Carcinoma	42 (10.6%)
Complications	
Intraprocedure	9 (2.3%)
Hemorrhage	5 (1.3%)
Perforation	3 (0.8%)
Infection	1 (0.2%)
Abdominal pain	0 (0.0%)
Postprocedure	56 (14.2%)
Hemorrhage	22 (5.6%)
Perforation	11 (2.8%)
Infection	11 (2.8%)
Abdominal pain	12 (3.0%)
Total	65 (16.5%)
Hospitalization (d)	
Postprocedure	6.6 ± 1.0 (2–21)
Total	8.4 ± 1.9 (4–27)

**Table 2:** Medical costs by category.

Category	USD
Hospitalization	178 (117–242)
Laboratory	452 (395–674)
Radiology	474 (422–790)
Nursing	191 (163–227)
Medication	2151 (1135–3465)
Anesthesia	385 (352–411)
Consumable items	1563 (1043–2667)
Operation	433 (379–796)
Covered by NHI	2757 (2039–3370)
Paid by patient	3070 (2882–3496)
Total	5927 (4615–9984)

**Table 3:** Major costs based on lesion location.

Classification	Rectum	Sigmoid colon	Descending colon	Transverse colon	Ascending colon	Cecum
	*n* = 86	*n* = 67	*n* = 78	*n* = 79	*n* = 69	*n* = 16
Medication	1843	1835	1866	2397	2474	2341
Consumable	1546	1412	1574	1801	1851	1790
Operation	425	381	439	443	399	406
Total	4917	4898	5755	6752	7148	6933

**Table 4:** Complications based on lesion location.

Complication	Location					
	Rectum	Sigmoid colon	Descending colon	Transverse colon	Ascending colon	Cecum
	*n* = 86	*n* = 67	*n* = 78	*n* = 79	*n* = 69	*n* = 16
Hemorrhage (*n* = 27)	7	5	4	5	6	0
Perforation (*n *= 14)	2	4	2	2	3	1
Infection (*n* = 12)	3	4	1	2	2	0
Abdominal pain (*n* = 12)	1	1	0	4	5	1

**Table 5:** Main costs based on lesion size, number, and pathology.

Category	Size (cm)			Number		Pathology			
	< 1,	1–5,	> 5,	1, *n*=298	>1, *n*=97	Polyp	Adenoma	Carcinoid	Carcinoma
	*n*=77	*n*=282	*n*=36			*n*=81	*n*=185	*n*=87	*n*=42
Medication	1705	2249	2583	2030	2528	1909	2197	2043	3259
Consumable items	1446	1691	1802	1419	1832	1326	1452	1465	2508
Operation	426	430	438	421	462	397	432	450	741
Total	5249	5921	6897	5773	6758	5674	5990	6252	9147

**Table 6:** Main costs based on surgical margins and complications.

Category	Surgical margin		Complication			
	Negative	Positive	Hemorrhage	Perforation	Infection	Abdominal pain
	*n*=331	*n*=64	*n*=27	*n*=14	*n*=12	*n*=12
Medication	2012	2988	2797	3086	2490	2385
Consumable items	1475	2460	1994	2418	1562	1649
Operation	404	718	502	717	425	432
Total	5626	8759	6135	8704	6056	5992

**Table 7:** Complications based on hospitalization days after the procedure.

Complication	Days			
	≤1	≤4	≤10	>10
Hemorrhage (*n*)	5	18	1	3
Perforation (*n*)	5	8	1	0
Infection (*n*)	2	8	1	1
Abdominal pain (*n*)	7	4	1	0

**Table 8:** Costs based on hospitalization days after the procedure.

Category	Hospitalization days (post-ESD)		
	≤4, *n*=84	4<n≤10, *n*=244	>10, *n*=67
Medication	1835	2479	3142
Consumable items	1390	1703	2004
Operation	408	426	579
Total	4811	5848	6925

**Table 9:** Costs based on defined outcomes.

Classification	Uncomplicated curative ESD of cancer	Uncomplicated curative ESD of HGIN	Uncomplicated noncurative ESD of cancer	Complicated ESD of cancer	Complicated ESD of other benign lesions
Medication	2557	2213	2748	3204	2717
Consumable items	1640	1502	1484	2276	1895
Operation	448	433	405	694	499
Total	6348	6185	8251	8739	7283

## Data Availability

The data and material are available from the corresponding author under reasonable request.
